# Preterm birth by vacuum extraction and neonatal outcome: a population-based cohort study

**DOI:** 10.1186/1471-2393-14-42

**Published:** 2014-01-22

**Authors:** Katarina Åberg, Mikael Norman, Cecilia Ekéus

**Affiliations:** 1 Department of Women’s and Children’s Health, Division of Reproductive Health, Karolinska Institutet, Retzius väg 13, 171 77 Stockholm, Sweden; 2 Department of Clinical Science, Intervention and Technology, Division of Pediatrics, Karolinska Institutet, Stockholm, Sweden; 3 Department of Women’s and Children’s Health, Division of Reproductive Health, Karolinska Institutet, Stockholm, Sweden

**Keywords:** Mode of delivery, Preterm delivery, Intracranial hemorrhage, Extracranial hemorrhage, Brachial plexus injury

## Abstract

**Background:**

Very few studies have investigated the neonatal outcomes after vacuum extraction delivery (VE) in the preterm period and the results of these studies are inconclusive. The objective of this study was to describe the use of VE for preterm delivery in Sweden and to compare rates of neonatal complications after preterm delivery by VE to those found after cesarean section during labor (CS) or unassisted vaginal delivery (VD).

**Methods:**

Data was obtained from Swedish national registers. In a population-based cohort from 1999 to 2010, all live-born, singleton preterm infants in a non-breech presentation at birth, born after onset of labor (either spontaneously, by induction, or by rupture of membranes) by VD, CS, or VE were included, leaving a study population of 40,764 infants. Logistic regression analyses were used to calculate adjusted odds ratios (AOR), using unassisted vaginal delivery as reference group.

**Results:**

VE was used in 5.7% of the preterm deliveries, with lower rates in earlier gestations. Overall, intracranial hemorrhage (ICH) occurred in 1.51%, extracranial hemorrhage (ECH) in 0.64%, and brachial plexus injury in 0.13% of infants. Infants delivered by VE had higher risks for ICH (AOR = 1.84 (95% CI: 1.09-3.12)), ECH (AOR = 4.48 (95% CI: 2.84-7.07)) and brachial plexus injury (AOR = 6.21 (95% CI: 2.22-17.4)), while infants delivered by CS during labor had no increased risk for these complications, as compared to VD.

**Conclusion:**

While rates of neonatal complications after VE are generally low, higher odds ratios for intra- and extracranial hemorrhages and brachial plexus injuries after VE, compared with other modes of delivery, support a continued cautious use of VE for preterm delivery.

## Background

Preterm birth is common [1] but still, the optimal mode of delivery of preterm infants is not known. Although neonatal outcomes in preterm infants delivered vaginally or by cesarean section (CS) [2-5] have been compared, there is no evidence to provide clear guidance on the method of choice [6]. Given the widespread assumption that assisted vaginal delivery could be harmful for fragile infants that are underweight and preterm, very few studies have addressed the use of vacuum extraction (VE) for preterm birth.

Delivery by VE is a common obstetrical procedure, and in many countries it has replaced the use of forceps. VE is used to terminate a protracted second stage of labor and as an intervention for fetal or maternal distress. VE requires vertex presentation, a fully dilated cervix and ruptured membranes [7]. A cesarean section, on the other hand, can be performed at any stage of labor and does not require prerequisites of this kind.

Most clinical guidelines do not recommend VE before 34 gestational weeks [8-10]. These recommendations are not based on results of randomized controlled trials, but rely on the observation that preterm infants are more likely than term infants to develop ICH, and on extrapolations from studies of term infants showing that VE is associated with an increased risk of ICH and other neonatal complications [11-17]. Only three studies have previously investigated the use and outcomes of VE in preterm births. The first was undertaken over 40 years ago and showed increased mortality and morbidity among preterm infants delivered by VE as compared with term infants delivered by VE [18]. The second study compared neonatal morbidity in preterm infants delivered vaginally with (n = 61) or without VE (n = 122), and found no differences in neonatal morbidity between the two groups [19]. The last study compared VE and forceps for preterm delivery (n = 64) [20]; the neonatal outcomes were similar in both groups. The available data are clearly untimely and hampered by limitations in power and, therefore, current knowledge on safety of preterm vacuum-assisted birth is unsatisfactory.

The aim of this study was to 1) describe the use of VE and compare it to rates of CS during labor in preterm deliveries in Sweden from 1999–2010, 2) characterize the distribution of perinatal risk factors associated with each mode of delivery, and 3) compare rates of neonatal intra- and extracranial hemorrhages, as well as occurrence of brachial plexus injury after preterm delivery by VE or CS during labor, using unassisted vaginal birth as a reference.

## Methods

This study was based on data from national data bases held by the Swedish National Board of Health and Welfare. The national registration number, assigned to each Swedish resident at birth, was used for individual record linkage. We used two registers: The Swedish Medical Birth Register (SMBR) that covers 99% of all births in Sweden, and The Swedish National Inpatient Register (IPR) that covers all public inpatient care. The SMBR includes prospectively collected information on maternal characteristics, reproductive history, and complications during pregnancy, delivery, and the neonatal period. The IPR includes data on each hospital admission and discharge.

### Study population

During the period of 1999–2010, there were 75,296 (6.2%) preterm births in Sweden. We **excluded** deliveries by CS before the onset of labor (n = 17,306), forceps (n = 257), or performed with both VE and CS (n = 125). We also excluded stillbirths (fetal deaths occurring before labor or intra partum) (n = 1,839), multiple births (n = 11,088), and births in breech presentation (n = 3,917). Thus, the final study population was restricted to all live-born, preterm singleton infants with a non-breech presentation at birth, delivered after a spontaneous or induced onset of labor followed by CS, vacuum extraction (VE), or by unassisted vaginal delivery (VD) before gestational week 37 + 0 days (N = 40,764). CS during labor was defined as abdominal delivery after the onset of labor, either spontaneously, by rupture of membranes, or by induction.

A number of independent variables were collected; the **maternal anthropometrics included**: age, height, and body mass index (BMI). BMI was calculated from measured height and weight obtained at the first antenatal care visit, which occurred before the 15^th^ week of gestation in more than 95% of the pregnancies. BMI was categorized into underweight (below 18.5 kg/m^2^), normal (18.5–24.9 kg/m^2^), overweight (25–29.9 kg/m^2^), obese (>29.9 kg/m^2^), or missing. **Parity** was categorized as primi- or multiparity. Information on complications during pregnancy and delivery were coded according to the International Classification of Diseases (ICD) Tenth Revision (1997 and onwards). The following **pregnancy complications were included**: *diabetes –* both gestational and types 1 and 2 (O24.0-9) *preeclampsia*- both hypertension, preeclampsia, and eclampsia (O10.0-O15.9). **Labor-related risk factors or covariates included***epidural analgesia* (EA; yes/no), and *induction of labor* (yes/no). **Indications for operative delivery** were classified into four major groups: *prolonged labor* (O62.0-2, O63.0-9), *signs of fetal distress* (O68.0-O68.1-9), preeclampsia, and *a non-occipitoanterior presentation* of the fetus (all presentations except occipitoanterior and breech, registered at birth). **Gestational age** (GA) for preterm infants was divided into three periods according to the World Health Organization: extremely preterm (before 28 weeks), very preterm (28–31 weeks) and moderately preterm (32–36 weeks). Furthermore, we also divided the preterm gestational period according to guidelines on instrumental delivery into either: less than 34 weeks (VE not recommended), and 34–36 weeks (VE may be used). GA was recorded in completed weeks, and was based on routine ultrasound dating performed at 17 to 18 postmenstrual weeks in 97-98% of all pregnant women. **Infant birthweight** was categorized as less than 1,500 grams, 1,500-2,000 grams, 2,001-2,500 grams, 2,501-3,000 grams, and 3,001-4,000 grams.

### Outcome variables

Neonatal diagnoses were classified according to the International Classification of Diseases (ICD) Tenth Revision (1997 and onwards), and identified/collected in the SMBR or in the IPR. The following neonatal outcomes (ICD codes) were assessed: *Intracranial laceration and hemorrhage due to birth injury* (P10), *intracranial non-traumatic hemorrhage of fetus and newborn* (P52), *convulsions of newborn* (P90), *other disturbances of cerebral status of newborn* (P 91), *subgaleal hematoma (*P12.2*), cephalhematoma* (P12.0), and *brachial plexus injury* (P14.0-3). The definitions of outcomes are described in detail in Table 1.

**Table 1:** Neonatal outcomes studied in 40 764 preterm infants

**Neonatal outcomes**			
**Outcome**	**Main ICD-code**	**ICD-subgroup**	
Intracranial bleeding	P10 Intracranial laceration and hemorrhage due to birth injury	10.0	Subdural hemorrhage due to birth injury
		10.1	Cerebral hemorrhage due to birth injury
		10.2	Intraventricular hemorrhage due to birth injury
		10.3	Subarachnoid hemorrhage due to birth injury
		10.4	Tentorial tear due to birth injury
		10.8	Other intracranial lacerations and hemorrhages due to birth injury
		10.9	Unspecified intracranial laceration and hemorrhage due to birth injury
	P52 Intracranial non-traumatic hemorrhage of fetus and newborn	52.0	Intraventricular (non-traumatic) hemorrhage, grade 1, Subependymal hemorrhage (without intraventricular extension)
		52.1	Intraventricular (non-traumatic) hemorrhage, grade 2, Subependymal hemorrhage with intraventricular extension
		52.2	Intraventricular (non-traumatic) hemorrhage, grade 3, Subependymal hemorrhage with both intraventricular and intracerebral extension
		52.3	Unspecified intraventricular (non-traumatic) hemorrhage of fetus and newborn
		52.4	Intracerebral (non-traumatic) hemorrhage of fetus and newborn
		52.5	Subarachnoid (non-traumatic) hemorrhage of fetus and newborn
		52.6	Cerebellar (non-traumatic) and posterior fossa hemorrhage of fetus and newborn
		52.8	Other intracranial (non-traumatic) hemorrhages of fetus and newborn
		52.9	Intracranial (non-traumatic) hemorrhage of fetus and newborn, unspecified
Neonatal cerebral dysfunction	P90 Convulsions of newborn	P90	Convulsions of newborn
	P91 Other disturbances of cerebral status of newborn	P91.0	Neonatal cerebral ischemia
		P91.1	Acquired periventricular cysts of newborn
		P91.2	Neonatal cerebral leukomalacia
		P91.3	Neonatal cerebral irritability
		P91.4	Neonatal cerebral depression
		P91.5	Neonatal coma
		P91.6	Hypoxic ischemic encephalopathy of newborn
		P91.8	Other specified disturbances of cerebral status of newborn
		P91.9	Disturbance of cerebral status of newborn, unspecified
Extracranial bleeding	P12 Birth injury to scalp	12.0	Cephalhaematoma due to birth injury
		12.2	Epicranial subaponeurotic haemorrhage due to birth injury
Neonatal nervous injury	P14 Birth injury to peripheral nervous system	14.0	Erb paralysis due to birth injury
		14.1	Klumpke paralysis due to birth injury
		14.2	Phrenic nerve paralysis due to birth injury
		14.3	Other brachial plexus birth injuries

Neonatal diagnoses of intracranial hemorrhages in preterm infants were mainly based on imaging of the brain using ultrasonography; however, some assessments of the brain at term-equivalent age were alternatively performed with CT and/or MRI. Imaging of the brain was performed on clinical indications only in cases born moderately or late preterm, whereas all very preterm infants (born before 32 weeks of gestation) were screened for intracranial lesions, even in asymptomatic infants. A diagnosis of convulsions included infants with clinical signs of convulsions and/or convulsions verified by EEG.

Statistical analysis was performed using proportions and odds ratios (OR) with a 95% confidence interval (CI) for neonatal complications in relation to mode of delivery, using unassisted VD as the reference group (SPSS 20.0 for Windows software package). Three models were used to assess the relationship between the different modes of delivery and the risk for neonatal complications: one crude, and two adjusted (Models 1 and 2). The included covariates have been shown previously to be related to instrumental deliveries, and were related to the outcomes in cross tabulations. In Model 1, we adjusted for the following confounders or covariates: maternal age, height, BMI, and parity, as well as infant year of birth, birthweight and GA. In Model 2, we added the indication for operative delivery and preeclampsia. The year of birth was entered as a continuous variable in accordance with a linear secular trend, and all other variables were entered as categories. Furthermore, a separate logistic regression analysis was performed to investigate severe ICH in relation to mode of delivery. Here, intraventricular hemorrhages grades 1–2 were excluded and the analysis was adjusted for GA only. We also conducted separated analyses on potential relationships between sex and ICH in relation to mode of delivery. Missing data were entered as a separate category in the analyses. The study was approved by the Regional Ethical Review Board in Stockholm, Dnr 2008/1322-31.

## Results

### Use of VE in relation to gestational age

**Figure 1:**
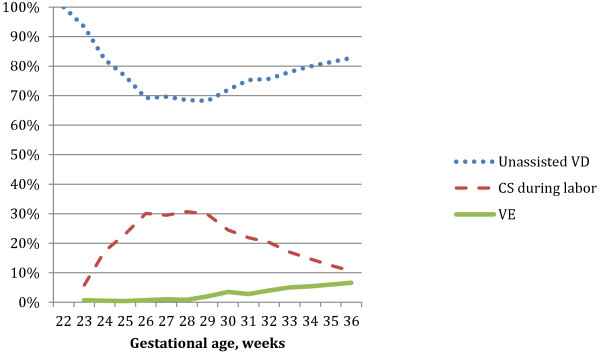
**Mode of delivery in relation to gestational age.** Figure 1 shows rates (%) of different modes of delivery in relation to gestational age (in completed weeks). The blue, dotted line represents unassisted vaginal deliveries, the red, dashed line represents cesarean sections performed after onset of labor, and the green line represents the vacuum extraction deliveries.

Among the 40,764 (54% of all) preterm deliveries included in this study, 2,319 (5.7%) preterm infants were delivered by VE, 5,505 (13.5%) by CS during labor, and 32,940 (80.2%) by VD. The rate of VE deliveries increased gradually with gestational age, Figure 1.

### Distribution of risk factors or covariates in relation to mode of delivery

Table 2 shows maternal and perinatal characteristics of the study population in relation to mode of delivery. The VE rate decreased with maternal height and 80% of the women who delivered by VE were primiparae, compared with 48% of those who underwent CS during labor (not in table). More than 45% of the women who delivered by VE had received epidural analgesia during labor compared with 22% of women with VD, and 12% with CS during labor (not in table). Given the association between GA and VE, infants delivered with VE had higher birthweights.

**Table 2:** Maternal, pregnancy, delivery, and infant characteristics by mode of delivery

	**Total**	**Unassisted vaginal delivery**	**CS during labor**	**Vacuum extraction**
	**N = 40,764**	**n = 32,940%**	**n = 5,505%**	**n = 2,319%**
**Maternal age, yrs**				
-19	1,063	85.0	10.3	4.7
20-24	6,130	85.6	9.3	5.0
25-29	12,845	82.2	11.6	6.2
30-34	12,767	79.8	14.2	6.0
35-39	6,380	76.4	18.6	5.0
>39	1,351	72.7	22.1	5.3
Missing	228	81.6	13.6	4.8
**Maternal height, cm**				
-155	2,481	75.6	18.0	6.4
156-160	6,538	79.9	14.3	5.8
161-165	10,336	81.0	13.0	6.0
166-170	10,129	81.8	12.5	5.7
>170	7,169	81.9	13.1	5.0
Missing	4,111	80.6	14.1	5.3
**Maternal BMI**				
Underweight	816	83.3	11.0	5.6
Normal	14,197	81.2	12.8	6.0
Overweight	6,124	79.0	15.2	5.8
Obese	2,944	76.4	19.4	4.1
Missing	16,683	81.7	12.6	5.7
**Parity**				
Primipara	18,120	80.0	11.6	8.4
Multipara	22,644	81.8	15.9	2.3
**Preeclampsia**				
Yes	1,833	64.2	28.3	7.5
No	38,931	81.6	12.8	5.6
**Diabetes**				
Yes	1,434	65.9	26.2	7.9
No	39,330	81.3	13.0	5.6
**Induced labor**				
Yes	6,372	73.8	19.9	6.3
No	34,392	82.1	12.3	5.6
**EA**				
Yes	8,894	80.9	7.3	11.8
No	31,870	80.8	15.2	4.0
**Gestational age, weeks**				
22-27	1,276	77.0	22.4	0.6
28-31	2,344	72.0	25.5	2.5
32-36	37,144	81.5	12.4	6.1
**Infant birthweight, g**				
< 1500	2,367	69.8	28.7	1.4
1501-2000	3,365	70.5	25.5	4.0
2001-2500	9,691	79.9	14.5	5.7
2501-3000	15,613	84.5	9.2	6.3
3001-4000	9,079	82.9	10.6	6.5
Missing	649	70.4	25.0	4.6

Population based cohort consisting of 40 764 preterm deliveries.

CS = caesarean section, BMI = Body Mass Index (weight in kilograms/height in meters^2^), EA = Epidural Analgesia.

The most common indication for VE was fetal distress (42%), followed by prolonged labor (25%). Having a non-occipitoanterior position (25%) or fetal distress (17%) were the most common indications for CS during labor, while only 3% in this group had a diagnosis of prolonged labor.

### Neonatal outcome in relation to gestational age

**Figure 2:**
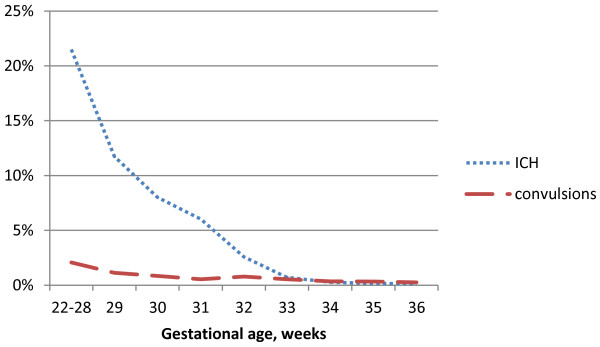
**Proportion (%) preterm infants diagnosed with intracranial hemorrhage (ICH) or convulsions by gestational age.** Figure 2 shows the proportions (%) of preterm infants diagnosed with ICH and neonatal convulsions in relation to gestational age (in completed weeks). The blue, dotted line represents intracranial hemorrhage (ICH) and the red, dashed line represents neonatal convulsions.

**Figure 3:**
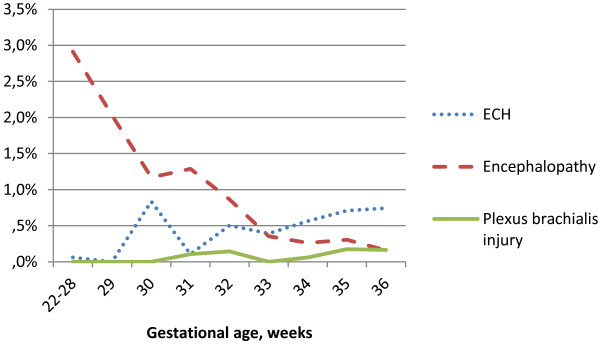
**Proportion (%) preterm infants diagnosed with extracranial hemorrhage (ECH), encephalopathy and brachial plexus injury by gestational age.** Figure 3 shows the proportions (%) of ECH, encephalopathy (ICD-code P91: other disturbances of cerebral status of newborn), and brachial plexus injury in relation to gestational age (in completed weeks). The blue, dotted line represents extracranial hemorrhage (ECH), the red, dashed line represents encephalopathy and the green line represents brachial plexus injury.

The proportion of preterm infants diagnosed with an ICH varied more than hundred-fold in relation to GA. It decreased from 21.5% among preterm infants born at 22–28 weeks of GA to 0.1% among those born after 36 weeks of gestation. The rates of neonatal convulsions among preterm infants decreased from 2.0% at 22–28 weeks to 0.25% among those born after 36 weeks of gestation, Figure 2. The proportion of preterm infants diagnosed with other disturbances of cerebral status (encephalopathy) decreased with GA, while proportion of infants with brachial plexus injuries or ECH increased slightly with GA, Figure 3.

### Neonatal outcome in relation to mode of delivery

To report outcome in relation to mode of delivery, the study cohort was divided according to the current guidelines on the use of VE as either preterm births occurring between 34–36 weeks of gestation (VE may be used), or those occurring before 34 gestational weeks (VE not recommended). In our cohort, 33,202 (81.4%) of all preterm births occurred at 34–36 gestational weeks, and 7,562 (18.6%) before 34 weeks of GA.

In Table 3, neonatal outcomes before and after 34 + 0 weeks of gestation are presented in relation to mode of delivery. Overall, seven preterm infants were classified as having an ICH due to birth injury, corresponding to a rate of 0.02%; and 612 infants were diagnosed with non-traumatic ICH, corresponding to a rate of 1.5%. Diagnoses of neonatal convulsions and other disturbances of neonatal cerebral status were rare, especially in infants at less than 34 weeks of GA, and occurred more frequently after VE and CS than after VD. Cephalic hematoma was the most frequent complication after VE of preterm infants (n = 72 or 3.1%), occurring much more often after VE than after CS (0.16%) and VD (0.49%). Subgaleal hemorrhage was less frequent, with a total of only18 cases. More than two thirds of those cases occurred in the VE group.

**Table 3:** Neonatal outcomes in preterm infants by mode of delivery and gestational age

	**Total**		**Unassisted vaginal delivery**		**Cesarean section during labor**		**Vacuum extraction**	
	**N = 40,764**		**n = 32,940**		**n = 5,505**		**n = 2,319**	
	**n**	**1/1000**	**n**	**1/1000**	**n**	**1/1000**	**n**	**1/1000**
**Intracranial hemorrhages**								
All	617	15.1	486	14.8	105	19.1	26	11.2
<34 weeks	564	74.6	451	79.0	95	59.4	18	71.7
34-36 weeks	53	1.6	35	1.3	10	2.6	8	3.9
**Convulsions**								
All	169	4.1	109	3.3	45	8.2	15	6.5
<34 weeks	74	9.9	48	8.4	23	14.4	3	12.0
34-36 weeks	95	2.9	61	2.2	22	5.6	12	5.8
**Other disturbances of cerebral status of newborn**								
All	168	4.1	98	3.0	53	9.6	17	7.3
<34 weeks	97	12.8	66	11.6	24	15.0	7	27.9
34-36 weeks	71	2.1	32	1.2	29	7.4	10	4.8
**Subgaleal and/or cephal hemorrhage**								
All	259	6.4	166	5.0	10	1.8	83	35.8
<34 weeks	24	3.2	11	1.9	5	3.1	8	31.9
34-36 weeks	235	7.1	155	5.7	5	1.3	75	36.3
**Brachial plexus injury**								
All	53	1.3	37	1.1	2	0.4	14	6.0
<34 weeks	3	0.4	3	0.5	0		0	
34-36 weeks	50	1.5	34	1.2	2	0.5	14	6.8

Table 4 shows rates (per 1000), crude and adjusted odds ratios for the outcomes by mode of delivery, and uses infants born by VD as the reference group. After adjustments, preterm infants born by VE had almost doubled OR for ICH, while those born by CS had no increased risk. Preterm infants delivered by VE also had 4.5 times higher OR for extracranial hemorrhages, while those delivered by CS had no increased risk. A total of 540 (88.2%) of the ICH diagnoses consisted of intraventricular bleedings (ICD-codes 52.0-52.3), the type of ICH most frequently seen in preterm infants. Of these, 438 (71.6%) were graded as mild or moderate (grades 1–2). After excluding grades 1–2 of intraventricular hemorrhages from the analyses, the OR for ICH was still significantly increased for infants delivered by VE (OR 2.58, CI 1.27-5.24), but not for those delivered by CS during labor (OR 1.15, CI 0.78-1.70) after adjustment for GA. There was no difference in ICH rates between preterm boys and girls delivered by VE.

**Table 4:** Logistic regression (odds ratios) for intra– and extracranial hemorrhages, convulsions and other cerebral complications, and brachial plexus injury in preterm infants exposed to different modes of delivery

**Mode of delivery**	**N**	**n**	**1/1000**	**Crude OR**	**AOR model 1**	**AOR model 2**
				**(95% CI)**	**(95% CI)**	**(95% CI)**
			**Intracranial hemorrhage**			
**Vaginal**	32,938	486	14.8	1.0	1.0	1.0
**CS during labor**	5,507	105	19.1	1.30 (1.05–1.61)	0.73 (0.58–0.92)	0.76 (0.58–0.98)
**VE**	2,319	26	11.2	0.76 (0.51–1.13)	2.05 (1.34–3.15)	1.84 (1.09–3.12)
**Total**	40,764	617	15.1			
			**Subgaleal– and/or cephalhematoma**			
**Vaginal**	32,938	166	5.0	1.0	1.0	1.0
**CS during labor**	5,507	10	1.8	0.36 (0.19–0.68)	0.42 (0.22–0.81)	0.36 (0.18–0.70)
**VE**	2,319	83	35.8	7.33 (5.61–9.57)	5.89 (4.46–7.78)	4.48 (2.84–7.07)
**Total**	4,0764	259	6.4			
				**Neonatal convulsions**		
**Vaginal**	32,938	109	3.3	1.0	1.0	1.0
**CS during labor**	5,507	45	8.2	2.48 (1.75–3.52)	1.95 (1.36–2.79)	1.42 (0.92–2.17)
**VE**	2,319	15	6.5	1.96 (1.14–3.37)	2.51 (1.44–4.38)	1.48 (0.73–3.01)
**Total**	40,764	169	4.1			
				**Other disturbances of neonatal cerebral status**		
**Vaginal**	32,938	98	3.0	1.0	1.0	1.0
**CS during labor**	5,507	53	9.6	3.26 (2.33–4.55)	2.27 (1.60–3.23)	1.61 (1.06–2.45)
**VE**	2,319	17	7.3	2.47 (1.48–4.15)	3.84 (2.24–6.56)	2.15 (1.09–4.27)
**Total**	40,764	168	4.1			
				**Plexus brachialis injury**		
**Vaginal**	32,938	37	1.1	1.0	1.0	1.0
**CS during labor**	5,507	2	0.4	0.32(0.08–1.34)	0.29 (0.07–1.23)	0.29 (0.07–1.26)
**VE**	2,319	14	6.0	5.40 (2.92–10.00)	6.45 (3.32–12.5	6.21 (2.22–17.4)
**Total**	40,764	53	1.3			

CS = cesarean section, VE = vacuum extraction.

**Model 1** is adjusted for year of birth, gestational age, parity, maternal age, height, BMI, and infant birthweight.

**Model 2** is also adjusted for indications for operative delivery.

The ORs for convulsions were almost doubled in both the VE and CS groups after adjustments for the variables in Model 1. However, further adjustment for indication for operative delivery decreased the odds and made the associations statistically insignificant. Other disturbances of the neonatal cerebral status were significantly increased (two to three times higher) both among infants delivered by VE, and by CS during labor, although the OR was higher in the VE group.

A total of 53 infants were diagnosed with brachial plexus injury. Of these, 14 were born by VE, corresponding to a rate in the VE group of 0.6% and an OR of 6.21 (95% CI: 2.22-17.4) in the fully-adjusted model. In contrast, infants delivered by CS had no increased risk for this complication. Among infants with brachial plexus injury, there were 11 cases of shoulder dystocia (ICD-code O66.0), of which five occurred in the VE group.

## Discussion

In this large cohort study of singleton, non-breech preterm births after onset of labor, we identify three clinically important findings related to mode of delivery: First, VE was used in 5.7% of preterm births, and despite recommendations of no use, 3.3% of preterm infants born before 34 gestational weeks were delivered by VE. Secondly, VE for preterm birth was used more frequently in shorter mothers, primiparae and among women treated with EA as pain relief during labor. Finally, and adjusting for potential confounders and covariates, preterm infants delivered by VE had almost doubled OR for ICH, four times higher OR for extracranial hemorrhage, as well as a 6-fold risk for brachial plexus palsy compared with those delivered by VD. Exclusion of intraventricular hemorrhage grades I-II (the most common form of ICH in preterm infants) from the analysis increased the OR for ICH after VE, indicating that severe bleedings were more common among preterm infants delivered by VE.

Although VE was related to significantly increased rates of ICH, it is not clear whether the extraction as such causes the injury, or if there is an underlying common pathway for both VE-assisted delivery and ICH, i.e., that the relationship is confounded by indication. Since the ORs for ICH were significantly higher in the VE group compared with both the CS and unassisted VD groups, whereas the ORs for other disturbances of cerebral status were slightly higher in both the VE and CS groups as compared with VD, different mechanisms may be involved in the development of these two complications. The forces by vacuum extraction could lead to significant vertical stress, which might be avoided with CS. In a case report of MRI findings after birth injuries among infants delivered by VE [21], it was suggested that vertical traction on the skull and brain may produce tentorial lacerations and rupture of intracranial veins. Another explanation for our findings of different outcomes after VE and CS could be that infants delivered by VE may have been exposed to contractions for a longer time than those delivered by CS. A protective effect of CS is indicated by lower ORs for ICH; however, the exposure to contractions as the sole explanation for the increased risks for ICH after VE is less likely, as the VD group – presumably the group exposed to the largest forces of labor – exhibited significantly lower odds for hemorrhagic complications compared with infants delivered with VE.

During the study period, the overall rate of ICH increased from 6% originally, up to 12% at the end of the period, most likely reflecting the increased access and use of ultrasonography among Swedish neonatologists in recent years. Improved ultrasound technology and image resolution may also have contributed to this development. Finally, we cannot exclude a contribution from misclassification: a large but normal choroid plexus could have been classified as a small subependymal hemorrhage by less experienced investigators. The finding that the diagnosis of small subependymal hemorrhage (without intraventricular extension; P52.0) increased most compared to other types of ICH during the study period (from 0.5% to 1.2%), supports these interpretations.

The overrepresentation of subgaleal hemorrhage and cephalhematoma after VE is less surprising, since earlier studies have stated the relation between these diagnoses and the use of VE. The risk of subgaleal hemorrhage seems to be unrelated to GA, as this study demonstrates rates similar to those in previous studies of infants born at term [12,13].

The present study showed that preterm infants delivered by VE had a 6- to 7-fold risk increase for injury to the plexus brachialis compared with unassisted VD. This injury is usually associated with large macrosomic infants and shoulder dystocia [22] and not to preterm birth. Our result emphasizes the importance of gentle maneuvers and avoiding application of excessive pressure or traction on the brachial plexus also when delivering the preterm infant, especially by VE.

The major strengths of this study were the large study population covering all preterm deliveries in Sweden during a period of twelve years, and the high quality of the registers, making it possible to analyze rare diagnoses and unusual events such as ICH in preterm infants delivered by VE. We were able to include data on risk factors, potential confounders, and outcomes collected independently from one another and without involving the study subjects, thus minimizing various types of bias (e.g., selection and recall bias). Moreover, antenatal and obstetric care is free of charge in Sweden, management routines as well as GA determinations are standardized, and 99% of births are delivered in public hospitals. This minimizes the risk for confounding by unmeasured socio-demographic factors. Another advantage was the inclusion of the main indications for VE and CS, enabling us to address the question of confounding by indication.

A major limitation of this study is that the registers do not contain detailed information about many important factors during the VE deliveries. For instance the registry does not provide specific information about the type of VE instrument used, level, position, and attitude of the fetal head in the pelvis when applying VE, location of placement of the vacuum cup, traction work, skill of the obstetrician, pressure exposure (duration and force), and cup detachments. The register does not provide information about confounders such as use of oxytocin and application of fundal pressure.

There is a general recommendation not to use VE before a GA of 34 weeks. According to the Royal College of Obstetricians and Gynecologists, there is insufficient evidence to establish the safety on VE deliveries in gestations between 34 weeks + 0 days and 36 weeks + 0 days [9]. Our results show that the use of VE is related to rare, but serious complications also between gestational weeks 34–36.

## Conclusion

The rates of serious birth injuries and complications are generally low, but preterm infants delivered by VE have higher odds ratios for intra- and extracranial hemorrhages and brachial plexus injuries than those delivered by CS during labor or by unassisted vaginal delivery. We therefore support a continued conservative/cautious use of VE in preterm deliveries. Furthermore, the possible causal relationship between mode of delivery and ICH needs to be further investigated.

## Abbreviations

BMI: Body mass index; CI: Confidence interval; CS: Cesarean section; CT: Computerized tomography; EA: Epidural analgesia; ECH: Extracranial hemorrhage; EEG: Electroencephalography; GA: Gestational age; ICH: Intracranial hemorrhage; LGA: Large for gestational age; OR: Odds ratio; SGA: Small for gestational age; VD: Vaginal delivery; VE: Vacuum extraction.

## Competing interests

The authors declare that they have no competing interest.

## Authors’ contributions

CE had the idea for the study, designed it and carried out the statistical analysis. KÅ participated in the design and wrote the first draft of the manuscript together with CE. MN contributed to the interpretation of the results and writing of the manuscript. All authors approved the final version of the submitted article.

## Pre-publication history

The pre-publication history for this paper can be accessed here:

http://www.biomedcentral.com/1471-2393/14/42/prepub
